# Periodontal Disease and Diabetes Mellitus in Pregnancy: An Overview of Systematic Reviews

**DOI:** 10.3390/dj14090539

**Published:** 2026-08-31

**Authors:** Albert Ramírez-Rámiz, María Dolores Rocha-Eiroa, Lluís Brunet-Llobet, Mar Muñoz-De Gea, Octavi Camps-Font, Jaume Miranda-Rius

**Affiliations:** 1 Department of Odontostomatology, Faculty of Medicine and Health Sciences, University of Barcelona, 08907 Barcelona, Spain; 2 Hospital Dentistry and Periodontal Medicine Research Group, Institut de Recerca Sant Joan de Déu (IRSJD), 08950 Barcelona, Spain; 3 Department of Dentistry, Hospital Sant Joan de Déu (HSJD), University of Barcelona, 08950 Barcelona, Spain; 4 School of Dentistry, Faculty of Medicine and Health Sciences, Bellvitge Campus, University of Barcelona, 08907 Barcelona, Spain; 5 Maxillofacial Dental Pathology and Therapy Research Group, Institut d’Investigació Biomèdica de Bellvitge (IDIBELL), 08908 Barcelona, Spain

**Keywords:** diabetes mellitus in pregnancy, gestational diabetes mellitus, maternal periodontal disease, periodontitis, gingivitis, adverse pregnancy outcomes, oral health education, women of reproductive age

## Abstract

**Background**: Periodontal disease has been increasingly associated with several systemic conditions, including diabetes mellitus. During pregnancy, both periodontitis and diabetes mellitus—including gestational and pre-existing forms (type 1 and type 2)—share common inflammatory and metabolic pathways that may influence maternal and neonatal outcomes. However, the consistency and strength of this association remain under debate. **Objective**: Our objective was to synthesize the available evidence from systematic reviews and meta-analyses on the association between maternal periodontal disease and diabetes mellitus during pregnancy, as well as its relationship with adverse pregnancy outcomes. **Methods**: An overview of systematic reviews was conducted following PRISMA recommendations. A comprehensive search was performed in PubMed, Scopus, Web of Science, and Cochrane CENTRAL from inception to the search date. A total of 161 records were identified, with 23 articles assessed in full text. Finally, nine systematic reviews were included, seven of which incorporated meta-analyses. Methodological quality was evaluated using AMSTAR 2, and risk of bias was assessed with ROBIS. Due to heterogeneity, a narrative synthesis was conducted. The degree of overlap among reviews was quantified using the corrected covered area (CCA). **Results**: The evidence consistently suggests a positive association between periodontal disease and diabetes mellitus during pregnancy, with increased risk reported in multiple meta-analyses, particularly for gestational diabetes mellitus. Periodontitis was also associated with adverse pregnancy outcomes, including preeclampsia, preterm birth, and low birth weight, although findings were not entirely homogeneous. The methodological quality of the included reviews was variable, predominantly low or critically low. A high level of overlap among reviews was observed (CCA = 15.2%). **Conclusions**: Current evidence supports an association between maternal periodontal disease, diabetes mellitus during pregnancy, and adverse maternal-fetal outcomes. However, these findings should be interpreted with caution due to methodological limitations and study overlap. Periodontal assessment in prenatal care and oral health education may be preventive strategies. Further studies are needed to clarify causality and evaluate these interventions.

## 1. Introduction

Periodontal disease (PD) is a chronic inflammatory condition affecting the supporting tissues of the teeth. In its most advanced form, periodontitis, it leads to irreversible attachment loss and alveolar bone resorption. Its global burden is substantial, with an estimated prevalence of approximately 50% for mild-to-moderate forms and 10.8% for severe disease [1].

From an etiopathogenic perspective, PD is associated with dysbiosis of the oral microbiota, characterized by a predominance of pathobionts over commensal microorganisms. This imbalance triggers an exaggerated host immune response, leading to the release of pro-inflammatory cytokines, C-reactive protein (CRP), and other mediators that contribute to both local tissue destruction and low-grade systemic inflammation. Ulceration of the periodontal pocket epithelium facilitates the hematogenous dissemination of microorganisms and bacterial products, such as lipopolysaccharide, amplifying systemic effects [2,3]. The detection of oral microorganisms and inflammatory mediators in placental tissues further supports the hypothesis of translocation from the oral cavity to the maternal–fetal environment [4].

Over the past decades, PD has been consistently associated with multiple systemic conditions, reinforcing the concept of a bidirectional relationship between oral and general health [2,5]. Pregnancy represents a particularly relevant physiological state in this context. Hormonal changes, especially increased levels of estrogen and progesterone, modulate the immune response and enhance susceptibility to periodontal inflammation, increasing both the prevalence and severity of disease in pregnant individuals [6].

A well-established bidirectional relationship also exists between PD and diabetes mellitus (DM). Chronic hyperglycemia exacerbates periodontal inflammation, while inflammatory mediators derived from PD—including TNF-α, IL-1β, and IL-6—interfere with glucose metabolism and promote insulin resistance [2,3]. This interrelationship has been recognized by international consensus reports from the International Diabetes Federation and the European Federation of Periodontology, which identify PD as both a complication of diabetes and a potential modifier of glycemic control [5]. During pregnancy, this inflammatory–metabolic axis acquires particular relevance, as gestation is characterized by progressive physiological insulin resistance that may be exacerbated by concomitant inflammatory conditions [2].

Hyperglycemia during pregnancy, whether due to pre-existing diabetes or gestational diabetes mellitus (GDM), represents a major public health concern. Evidence from systematic reviews suggests a significant association between PD and GDM [7], while recent observational studies in postpartum populations support this relationship in real-world clinical settings [8]. Globally, GDM affects approximately 16% of pregnancies, potentially up to one in five according to data from the International Diabetes Federation [9]. This condition is associated with an increased risk of maternal and perinatal complications, including preeclampsia, preterm birth, low birth weight, and long-term metabolic alterations in the offspring [6,9].

Emerging evidence suggests that periodontal inflammation may contribute to a systemic pro-inflammatory state that adversely influences pregnancy outcomes. The identification of inflammatory mediators and oxidative stress markers in placental tissues further reinforces the biological plausibility of a complex interplay between periodontitis, diabetes in pregnancy, and adverse pregnancy outcomes [2,3,6]. However, methodological heterogeneity and inconsistencies across studies limit the overall interpretation of this association [7,8].

In this context, a high-level synthesis of the available evidence is warranted. Overviews of systematic reviews represent an appropriate methodological approach to integrate findings from multiple reviews and meta-analyses, enabling the assessment of consistency, quality, and robustness of evidence in complex clinical scenarios [10].

The aim of this overview is to synthesize the evidence from systematic reviews on the association between maternal periodontal disease and diabetes mellitus during pregnancy, as well as its relationship with adverse maternal and neonatal outcomes.

## 2. Materials and Methods

An overview of systematic reviews was conducted following the Preferred Reporting Items for Systematic Reviews and Meta-Analyses (PRISMA) guidelines [10]. The study protocol was registered in PROSPERO (CRD420261353938).

### 2.1. Eligibility Criteria

The research question was formulated using the PECO strategy (Population, Exposure, Comparator, and Outcomes). The population included pregnant women with gestational diabetes mellitus (GDM) or pre-existing diabetes. The exposure was periodontal disease (PD), compared with the absence of PD. Outcomes included glycemic control and adverse maternal and neonatal outcomes. The research question was: “*Is periodontal disease in pregnant women with diabetes associated with an increased risk of adverse pregnancy outcomes compared with diabetic pregnant women with preserved periodontal health?*”

Systematic reviews, with or without meta-analysis, conducted in humans and evaluating the association between periodontal disease and diabetes during pregnancy were included, provided they reported at least one relevant clinical outcome. Studies were excluded if they were primary studies (cohort, case–control, or cross-sectional), narrative reviews, editorials, letters, conference abstracts, animal studies, or if they did not address the interaction between periodontal disease, diabetes, and pregnancy. No language or publication date restrictions were applied.

### 2.2. Information Sources and Search Strategy

A comprehensive literature search was conducted in PubMed, Scopus, Web of Science, and Cochrane CENTRAL up to May 2026. The main search terms included “periodontal disease” (PD), “periodontitis”, “gingivitis”, “dysbiosis oral”, “diabetes mellitus”, “gestational diabetes mellitus” (GDM), “pregnancy”, “pregnancy complications”, “pregnant women”, “systematic review”, and “meta-analysis”. Boolean operators (AND/OR) were used to combine the search terms. The search strategy was developed for PubMed and subsequently adapted for the remaining databases. Reference lists of all included studies were manually screened to identify additional relevant reviews. Two reviewers independently performed the search (A.R.-R., M.D.R.-E.). The search strategy was structured into four conceptual blocks: periodontal disease, diabetes mellitus, pregnancy complications, and study design (systematic reviews and meta-analyses). Controlled vocabulary (MeSH terms) and free-text terms were combined within these blocks and adapted for each database. In addition, the reference lists of included studies were manually screened to identify potentially relevant publications.

### 2.3. Study Selection

Study selection was performed in two phases. First, two reviewers (A.R.-R., M.D.R.-E.) independently screened titles and abstracts, followed by full-text assessment of potentially eligible studies. Disagreements were resolved by consensus or by consulting a third reviewer (J.M.-R.). The selection process was documented using a PRISMA flow diagram. Inter-examiner agreement was calculated using Cohen’s kappa coefficient.

### 2.4. Data Extraction

Data extraction was conducted independently by two reviewers (A.R.-R., M.D.R.-E.) using a standardized form. Extracted data included author, year, country, journal, study type, number of included studies, population, objectives, and main findings. Data were summarized in tabular form.

The degree of overlap among reviews was quantified using the corrected covered area (CCA) [11].

### 2.5. Quality Assessment and Data Synthesis

The methodological quality of the included reviews was assessed using AMSTAR 2, which evaluates 16 domains and classifies overall quality as high, moderate, low, or critically low based on critical weaknesses [12]. The assessment was performed independently by two reviewers (A.R.-R., M.D.R.-E.), with discrepancies resolved by consensus.

Due to clinical and methodological heterogeneity, no additional meta-analysis was conducted. Instead, findings were synthesized narratively, considering the magnitude and consistency of effects, as well as the methodological quality of the included reviews. To identify design flaws, the ROBIS tool was used in order to detect the risk of bias more accurately. Quantitative data were interpreted descriptively within the overall context of the available evidence.

## 3. Results

A total of 161 records were identified across the four databases: PubMed (n = 31), Cochrane Library (n = 5), Scopus (n = 79), and Web of Science (n = 46). No additional studies were identified through other sources. After removing duplicates (n = 63), 98 records were screened by title and abstract.

**Figure 1:**
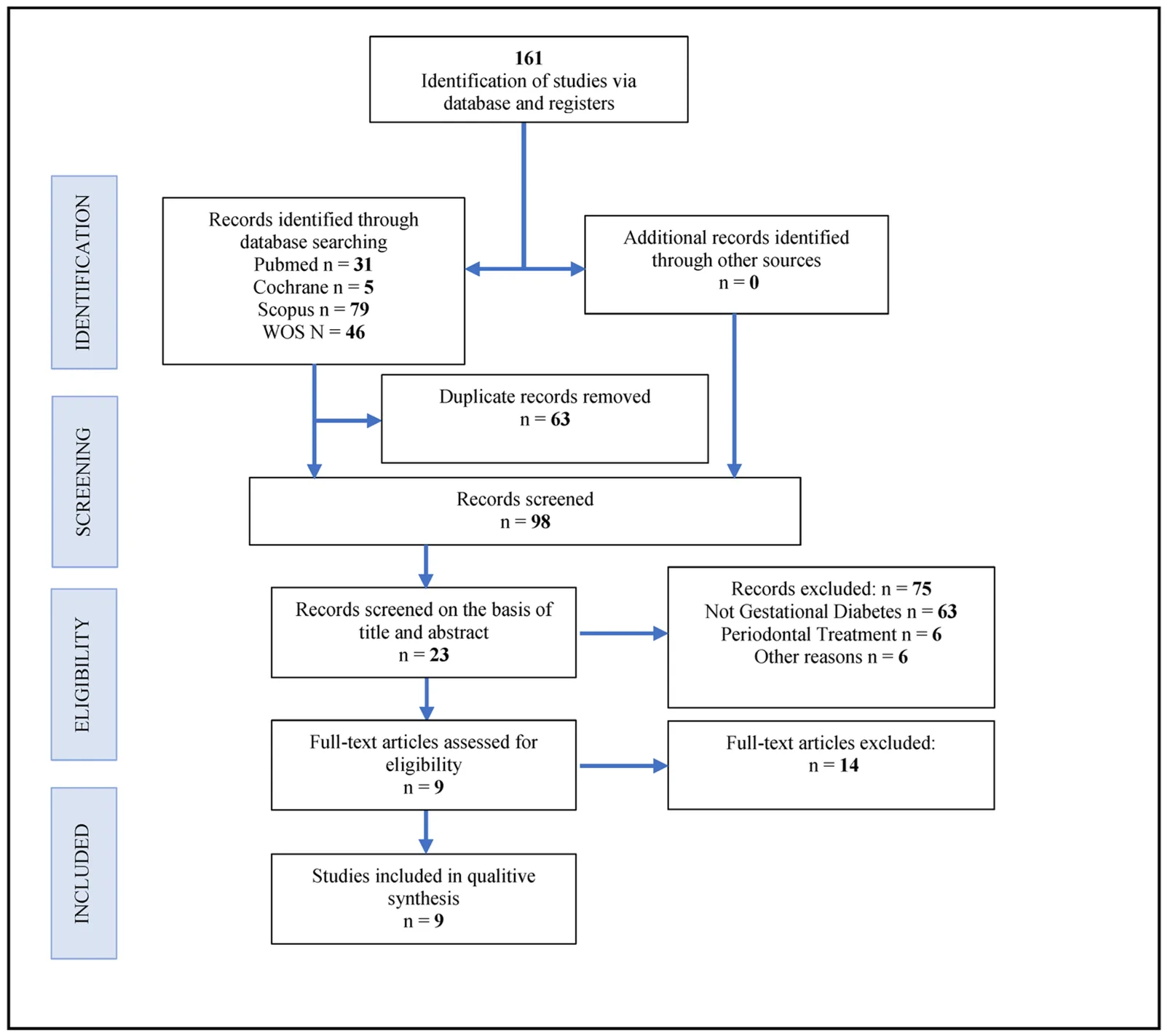
PRISMA 2020 Flow diagram for new systematic reviews that included searches of databases and registers only.

Of these, 23 studies were considered potentially eligible for full-text assessment. Following detailed evaluation, 14 articles were excluded for not meeting the inclusion criteria, resulting in the inclusion of nine systematic reviews in the qualitative synthesis. The study selection process is summarized in the PRISMA flow diagram (Figure 1). Inter-reviewer agreement during study selection was high (Cohen’s kappa = 0.85).

The included reviews, published between 2007 and 2026, showed considerable heterogeneity in study design, sample size, and outcomes assessed. Seven of the nine reviews included meta-analyses evaluating the association between periodontal disease (PD) and gestational diabetes mellitus (GDM), as well as its impact on adverse pregnancy outcomes. The number of primary studies included per review ranged from 8 to 67. Detailed characteristics are presented in Table 1 [13,14,15,16,17,18,19,20,21]. The corrected covered area (CCA) was 15.2%, indicating a high degree of overlap among the included reviews.

### 3.1. Association Between Periodontal Disease and Diabetes in Pregnancy

The periodontal health varied among the included reviews according to the classification systems and diagnostic thresholds used in the original studies. Although most reviews supported a positive association between PD and GDM, some reported inconclusive findings, mainly due to methodological heterogeneity and differences in diagnostic criteria [13,14,15,18,19,20]. Although the magnitude of the effect varied across studies, the direction of the association remained consistent. Meta-analysis reported a significantly higher risk of GDM in women with periodontitis, whereas no consistent association was observed for gingivitis [14]. In addition, women with GDM showed higher levels of periodontal inflammation, including increased bleeding on probing (BOP) and probing pocket depth (PPD), without significant differences in clinical attachment level (CAL) [14,15]. In general, periodontal health referred to the absence of clinical signs of gingival inflammation and destructive periodontal disease (Table 1).

**Table 1:** Summary of the description of the included reviews.

Authors	Study Nº Articles	Population & Aim	OR/RR	Heterogeneity (I^2^) %	Outcomes
**Yang et al.** **2026 [13]**	SR + MA 21	8627 Quantitative assessment of PD prevalence in pregnancy and its association with GDM.	OR:1.69	71.40%	PD was significantly associated with an increased risk of GDM in the overall analysis.
**Giancotti et al.** **2026 [14]**	SR + MA 14	3800 Association between PD and GDM and implications for preventing GDM and APOs.	OR: 2.10	46%	Significant association between GDM and P.
**García-Martos et al. 2025 [15]**	SR + MA 11	2032 Evaluate association between PD & development of GDM.	OR: 1.83	40.83%	Significant association between PD and GDM.
**Sadiqa et al.** **2025 [16]**	SR 38	----- Association between PD, APOs, and the underlying mediators and mechanisms.	No MA	----	Consistent association between PD with GDM, PE, PTB, and LBW outcomes.
**Karimi et al.** **2023 [17]**	SR + MA 67	---- Association between PD and the risk of APOs.	GDM: 1.39 LBW: 2.19 PE: 1.43 PTB: 1.10 PROM: 1.25	GDM: 49.67% LBW: 0.00% PE: 82.64% PTB: 88.14% PROM: 61.73%	PD is associated with an increased risk of GDM, PE and LBW.
**León-Ríos et al.** **2022 [18]**	SR 8	1897 Association between PD and GDM risk in pregnancy.	No MA	----	Positive association; heterogeneous findings.
**Abariga & Whitcomb** **2016 [19]**	SR + MA 10	5724 Association between P and GDM and GDM risk.	OR: 1.66	50.5%	PD associated with increased GDM risk.
**Esteves Lima et al.** **2016 [20]**	SR + MA 8	5407 Evidence on the association between P and GDM in pregnant women.	OR: 1.67	0%	Positive association between P and GDM; inconsistent case-control findings.
**Xiong et al.** **2007 [21]**	SR + MA 44	---- PD, APOs, and underlying mechanisms.	OR: 9.11	----	Association observed in 29 studies; high heterogeneity.

I^2^: percentage of total variation across studies attributable to heterogeneity rather than chance. APOs: Adverse pregnancy outcomes; GDM: Gestational diabetes mellitus; LBW: Low birth weight; MA: Meta-Analysis; PD: Periodontal disease; P: Periodontitis; PE: Pre-eclampsia; PROM: Premature Rupture of Membranes; PTB: Preterm birth.

### 3.2. Adverse Pregnancy Outcomes

Several reviews reported an association between PD and adverse maternal and neonatal outcomes, including preeclampsia, preterm birth, low birth weight, intrauterine growth restriction, and miscarriage [17,21]. However, these findings were not entirely consistent, reflecting heterogeneity in study design and diagnostic criteria.

From a mechanistic perspective, some reviews highlighted systemic inflammation as a key link between PD and pregnancy complications. Elevated inflammatory mediators and the systemic dissemination of periodontal pathogens may contribute to metabolic dysfunction and placental alterations [16,17].

Evidence on periodontal treatment suggests a potential reduction in adverse pregnancy outcomes, although results remain inconsistent due to the limited number of clinical trials and variability among studies [21] (Table 1).

### 3.3. Quality Assessment of Included Reviews

**Figure 2:**
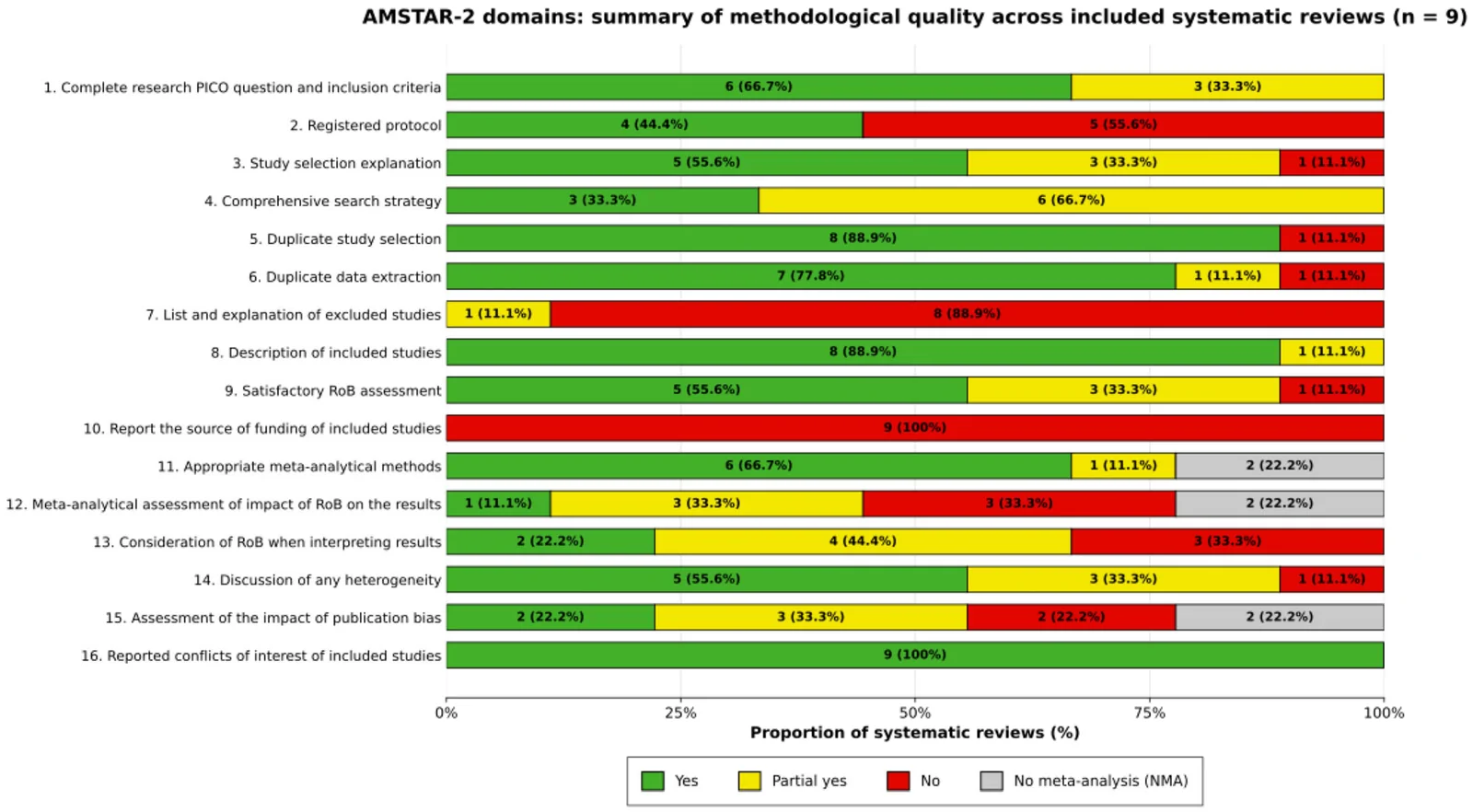
Graphical summary of the AMSTAR-2 results. Each bar represents the proportion (%) of reviews (n = 9) that fully comply with the domain (Yes), partially comply (Partial yes), do not comply (No) or are not applicable due to the absence of a meta-analysis (NMA). Abbreviations: RoB, risk of bias; NMA, no meta-analysis.

The methodological quality of the included reviews, assessed using AMSTAR 2 (Figure 2), showed considerable variability, with most reviews rated as low or critically low quality.

None of the reviews fulfilled all 16 AMSTAR 2 domains. Six reviews were classified as critically low quality [15,16,17,18,19,21] and three as low quality [13,14,20]. Although most reviews adequately described the included studies, re-ported conflicts of interest, and performed duplicate study selection and data extraction, important methodological limitations were common. These included the absence of protocol registration in several reviews, the lack of a list of excluded studies, limited assessment of publication bias, and insufficient consideration of the impact of risk of bias on the reported findings. None of the reviews reported the funding sources of the included studies. Overall, these methodological shortcomings warrant cautious interpretation of the available evidence.

Most reviews presented a well-defined research question, generally aligned with the PECO framework, and applied duplicate and independent study selection and data extraction, supporting internal validity.

Risk of bias was evaluated using the ROBIS tool [22]. Although many reviews used the Newcastle–Ottawa scale to assess observational studies, only six explicitly incorporated risk of bias into the interpretation of their findings. Overall, most reviews were judged to have a high risk of bias, mainly due to limitations in study identification and selection processes and the absence of justified exclusion lists.

Only two reviews were classified as having low overall risk of bias, corresponding to better adherence to critical AMSTAR 2 domains [13,20] (Table 2). Inter-reviewer agreement for quality assessment was high (Cohen’s kappa = 0.82).

## 4. Discussion

Since the pioneering studies by Offenbacher et al. [23,24], which first proposed an association between periodontal disease (PD) and adverse pregnancy outcomes, substantial evidence has accumulated supporting the link between periodontal health and systemic conditions. PD is now recognized as a potentially modifiable risk factor associated with low-grade systemic inflammation, which may contribute to both diabetes mellitus and pregnancy complications [21]. In the literature, the prevalence of periodontitis among pregnant women in industrialized countries has been reported to range from 20% to 50%. This variability is partly explained by differences in the diagnostic criteria used to define periodontitis [25].

The findings of this overview indicate a consistent association between maternal PD and diabetes mellitus during pregnancy, particularly GDM. Most included systematic reviews reported a positive relationship, with periodontitis associated with an increased risk of GDM [13,14,15,18,19]. Previous meta-analyses have estimated a 60% to twofold increase in risk, with more recent studies reporting odds ratios around 2.0 [13,14,19]. However, this association is not fully conclusive, largely due to methodological heterogeneity, variability in diagnostic criteria, and differences in study design. Inconsistent findings have been reported, especially in studies with methodological limitations or inadequate control of confounders such as maternal age, smoking, socioeconomic status, and ethnicity [17,19,20,26].

Both pregnancy and diabetes are characterized by an enhanced inflammatory state, which may obscure the independent contribution of periodontal inflammation to metabolic dysregulation. Chronic, low-grade inflammation—although not always causing immediate tissue damage—may contribute to the development of metabolic and obstetric complications [27]. Conversely, evidence from longitudinal studies remains insufficient to confirm whether GDM, given its transient nature, can initiate or exacerbate PD, limiting conclusions regarding a fully bidirectional relationship [17,20].

### 4.1. Biological Mechanisms Linking Periodontal Disease and Diabetes in Pregnancy

Several plausible mechanisms may explain this association. Periodontal inflammation induces a systemic pro-inflammatory state characterized by elevated cytokines such as TNF-α, IL-1β, and IL-6, as well as C-reactive protein, all of which may interfere with insulin signaling and promote insulin resistance [16,28,29].

Pregnancy-related physiological changes further contribute to this process. Hormonal fluctuations can modify the oral biofilm, increase vascular permeability, and enhance gingival inflammation [17,30,31,32]. Additionally, alterations in salivary flow and pH during pregnancy may promote the growth of periodontal pathogens, such as *Prevotella intermedia* [33,34]. The oral microbiota may therefore become more unstable in the presence of systemic or local alterations [35].

At the molecular level, inflammatory biomarkers in gingival crevicular fluid have been proposed as potential early indicators of metabolic dysfunction during pregnancy, although their clinical applicability requires further validation [14]. Increased levels of MMP-8 and MMP-9 in early pregnancy have been associated with both periodontitis severity and GDM [17,36]. Furthermore, the detection of oral microorganisms and inflammatory mediators in placental tissues supports the hypothesis of hematogenous dissemination, suggesting a direct role of oral infection in pregnancy complications [4].

### 4.2. Periodontal Disease and Adverse Pregnancy Outcomes

Evidence from the included reviews suggests that PD is not only associated with GDM but may also contribute to adverse maternal and neonatal outcomes, including preeclampsia, preterm birth, and low birth weight [17,21,37]. Indeed, the PD criteria applied by the researchers, according to the various indices, can alter the evidence of the relationship between periodontitis and preterm birth [25].

Several biological mechanisms have been proposed to explain these associations, including the systemic dissemination of periodontal pathogens and inflammatory mediators, which may contribute to placental inflammation and alterations in the maternal–fetal environment. Although the magnitude and consistency of these associations vary across studies, the available evidence supports a biologically plausible relationship between periodontal inflammation and adverse maternal and neonatal outcomes [21,25,35].

This relationship may be explained by an oral–systemic–placental axis. Periodontal pathogens such as *Porphyromonas gingivalis*, *Fusobacterium nucleatum*, and *Tannerella forsythia*, along with their endotoxins, have been implicated in pregnancy complications through hematogenous dissemination to the fetal–placental unit [17,29], potentially triggering local inflammatory responses and impairing placental function.

Meta-analytic evidence supports these associations, reporting increased risks of GDM (RR ≈ 1.39), low birth weight (RR ≈ 2.19), and preeclampsia (RR ≈ 1.43) in women with PD [17]. However, findings are not entirely consistent. Some reviews report only moderate or weak associations with preterm birth and low birth weight, likely reflecting methodological heterogeneity and risk of bias [38,39,40].

Overall, the evidence suggests that maternal PD may act as a modifier of the gestational inflammatory environment, increasing susceptibility to complications. However, the variability of findings highlights the need for cautious interpretation.

### 4.3. Clinical Implications and Multidisciplinary Approach

From a clinical perspective, these findings support the integration of periodontal assessment into prenatal care. Periodontal screening may improve both oral health and metabolic control in pregnant women [14,29,37]. A multidisciplinary approach involving dentistry, obstetrics, and endocrinology is therefore essential [40,41]. In addition, pregnancy-related hormonal changes may exacerbate gingival inflammation and contribute to the development of pregnancy-associated gingival lesions, including pyogenic granulomas (“pregnancy tumors”), which may further affect oral health and quality of life during pregnancy [6,42]. Moreover, greater awareness among healthcare professionals is needed regarding the impact of periodontal health on pregnancy outcomes. Strengthening interdisciplinary collaboration may improve prevention and early management strategies.

### 4.4. Oral Health Education and Prevention

From a public health perspective, PD represents a modifiable risk factor with potential implications for the prevention of GDM and adverse pregnancy outcomes. Periodontal health education in pregnant women is therefore a key strategy to translate scientific evidence into clinical practice.

**Figure 3:**
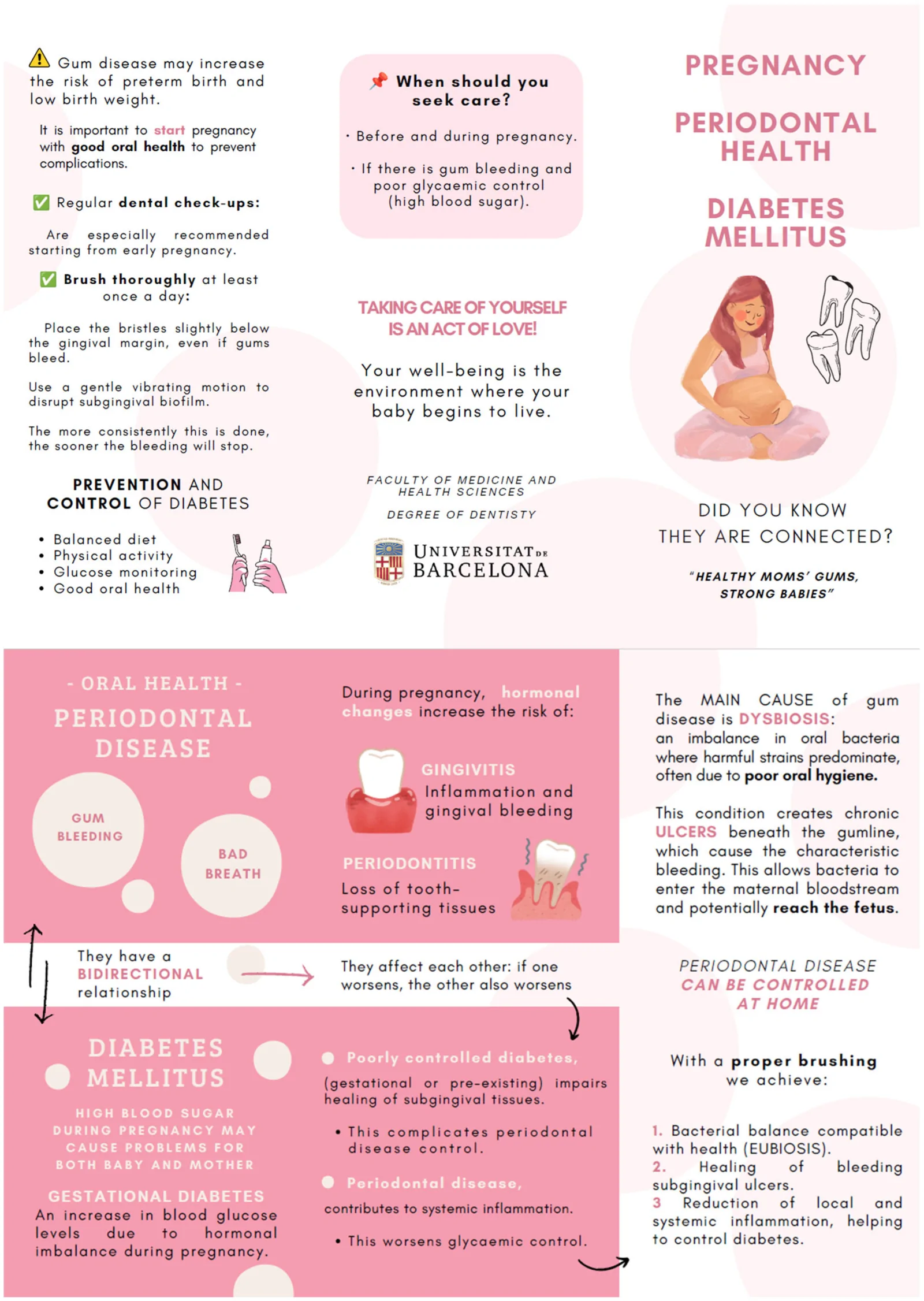
Educational leaflet promoting periodontal health in pregnant women, designed to support preventive strategies and improve oral health awareness.

Educational initiatives, including structured oral health promotion programs and awareness materials targeting pregnant women, may support preventive strategies and improve adherence to oral hygiene practices [42,43]. An example of this approach is illustrated in Figure 3. These strategies, particularly structured interventions aimed at improving oral hygiene behaviours, have shown promising results in enhancing patient adherence and reducing modifiable risk factors [42]. Initiatives such as educational materials targeting women of reproductive age may enhance awareness and promote early prevention.

In this context, structured self-care programs based on clinical models, such as the TIME wound-healing model (Tissue, Infection/Inflammation, Moisture, and Edges), may improve the management of chronic inflammatory conditions, including PD [42]. Although these strategies show potential, their effectiveness in reducing maternal–fetal complications requires further evaluation through well-designed studies [19,29,44].

### 4.5. Limitations

Despite the overall consistency in the direction of the reported associations, several limitations should be considered when interpreting the findings of this overview. First, substantial heterogeneity was observed across the included reviews regarding the diagnostic criteria used for periodontal disease, particularly for the definition and classification of periodontitis. Variations in case definitions, periodontal parameters, and disease severity thresholds may have influenced the reported estimates and limit direct comparisons among studies. Furthermore, substantial heterogeneity in the reporting of periodontal, glycemic, and pregnancy-related outcomes precluded additional quantitative synthesis. Therefore, the findings were narratively summarized based on the pooled estimates reported by the original authors whenever available.

Second, important methodological differences were identified among the included systematic reviews. The AMSTAR 2 assessment showed that most reviews were classified as low or critically low quality, mainly due to shortcomings in critical domains such as protocol registration, comprehensiveness of search strategies, risk of bias assessment, and evaluation of publication bias. Likewise, the ROBIS assessment identified concerns related to study identification and selection procedures, as well as the incorporation of risk-of-bias assessments into the interpretation of findings.

Another important limitation was the considerable overlap among the included systematic reviews (CCA = 15.2%). This finding indicates that several reviews relied on many of the same primary studies, increasing the possibility of redundancy and potentially amplifying the apparent consistency of the evidence [2,28]. In addition, differences in comparator groups, including the use of normoglycemic pregnant women or women with periodontal health as controls, may have contributed to variability in the reported findings.

Interpretation of the available evidence is also complicated by the presence of residual confounding factors in the original studies, including maternal age, obesity, smoking status, socioeconomic conditions, ethnicity, and differences in healthcare access [17,19,20,21,26]. These factors may influence both periodontal status and metabolic outcomes during pregnancy and were not consistently controlled across studies.

Furthermore, outcome reporting varied considerably among reviews. Definitions of periodontal health, glycemic control, gestational diabetes mellitus, and adverse pregnancy outcomes were not uniform, limiting comparability and precluding quantitative synthesis within this overview. Similarly, mean clinical parameters and glycemic measurements were not consistently reported across the included reviews.

Finally, evidence regarding the effectiveness of periodontal treatment and oral health educational interventions during pregnancy remains limited and inconclusive. Although some studies suggest potential benefits for periodontal status and maternal health, robust evidence demonstrating a consistent reduction in adverse maternal or neonatal outcomes is still lacking [21,40,42,43,44,45].

Therefore, while the available evidence supports an association between periodontal disease, diabetes mellitus in pregnancy, and adverse pregnancy outcomes, the methodological limitations of the existing literature warrant cautious interpretation and highlight the need for higher-quality research.

### 4.6. Future Research Directions

Future research should focus on well-designed prospective cohort studies and randomized controlled trials using standardized periodontal diagnostic criteria and internationally accepted definitions of gestational diabetes mellitus. Greater methodological consistency would facilitate more reliable comparisons across studies and improve the overall quality of evidence [18,19,44].

In addition, future investigations should explore the role of inflammatory biomarkers, periodontal parameters, and oral microbiome profiles as potential predictors of metabolic and obstetric complications during pregnancy [16,17,36]. Standardized reporting of glycemic outcomes, periodontal measures, and maternal-neonatal outcomes would facilitate evidence synthesis and improve clinical interpretation across studies.

Particular attention should be paid to the control of important confounding factors, including maternal age, obesity, smoking habits, socioeconomic status, and ethnicity, which may influence both periodontal and metabolic conditions during pregnancy [17,19,20,26].

Further research is also needed to determine whether periodontal therapy before or during pregnancy can improve glycemic control and reduce adverse pregnancy outcomes. Although some studies suggest potential benefits, the current evidence remains inconsistent and insufficient to support definitive conclusions [21,40,45].

Finally, the effectiveness of structured oral health education programs should be assessed in adequately powered interventional studies with long-term follow-up. Such investigations could help determine whether preventive educational strategies contribute to improving oral health behaviours, periodontal outcomes, and maternal–fetal health [42,43,44].

## 5. Conclusions

The evidence synthesized in this overview supports a consistent association between periodontal disease and diabetes mellitus in pregnancy, particularly gestational diabetes mellitus. Women affected by periodontal disease appear to present an increased risk of GDM, while women with diabetes during pregnancy frequently exhibit poorer periodontal status and increased periodontal inflammation.

The available evidence also suggests an association between periodontal disease and adverse pregnancy outcomes, including preeclampsia, preterm birth, and low birth weight. However, the strength of these associations varies across studies, and methodological limitations prevent definitive conclusions regarding causality.

Evidence regarding the impact of periodontal disease on glycemic control during pregnancy remains heterogeneous and less conclusive. Moreover, the low methodological quality of many systematic reviews, together with the substantial overlap among primary studies, warrants cautious interpretation of the available findings.

From a clinical and public health perspective, the findings support the integration of periodontal assessment into prenatal care and highlight the potential value of oral health education as part of preventive maternal healthcare. Future well-designed prospective studies and randomized controlled trials are needed to clarify causal relationships and to determine the effectiveness of periodontal and educational interventions in improving maternal and neonatal outcomes.

## Figures and Tables

**Table 2:** Risk-of-bias assessment of the included systematic reviews according to the ROBIS tool.

Review	Study Eligibility Criteria	Identification and Selection of Studies	Data Collection and Study Appraisal	Synthesis and Findings	Risk of Bias
**Yang et al.** **2026 [13]**	Low	Low	Low	Low	Low risk
**Giancotti et al.** **2026 [14]**	Low	High	Moderate	Moderate	High risk
**García-Martos et al. 2025 [15]**	Low	High	Low	Moderate	High risk
**Sadiqa et al.** **2025 [16]**	Low	High	Moderate	High	High risk
**Karimi et al.** **2023 [17]**	Low	High	Low	Moderate	High risk
**León-Ríos et al.** **2022 [18]**	Low	High	Moderate	High	High risk
**Abariga et al.** **2016 [19]**	Low	High	Low	Moderate	High risk
**Esteves Lima et al. 2016 [20]**	Low	Low	Low	Low	Low risk
**Xiong et al.** **2007 [21]**	Low	High	High	High	High risk

## Data Availability

The datasets generated and analyzed during the current study are available from the corresponding author, upon reasonable request.
